# Reasons for non-participation in a primary care-based physical activity trial: a qualitative study

**DOI:** 10.1136/bmjopen-2016-011577

**Published:** 2016-05-23

**Authors:** S Attwood, K L Morton, J Mitchell, M Van Emmenis, S Sutton

**Affiliations:** 1 MRC Epidemiology Unit, Institute of Metabolic Science, University of Cambridge School of Clinical Medicine, Cambridge, UK; 2 Behavioural Science Group, Primary Care Unit, Institute of Public Health, University of Cambridge, Cambridge, UK

**Keywords:** physical activity, equity, general practice

## Abstract

**Objectives:**

To explore reasons for non-participation in a primary care-based physical activity trial and understand how these may contribute to recruitment of non-representative research samples. We also aimed to elicit non-participants’ own recommendations for enhancing trial uptake in primary care.

**Design:**

Semistructured telephone interviews with non-participants to a randomised controlled trial of a very brief intervention for promoting physical activity conducted in primary care (the Very Brief Interventions trial), with thematic analysis of interview transcripts.

**Setting:**

5 general practice (GP) surgeries in the East of England, UK.

**Participants:**

Interviews were completed with 10 female and 6 male non-participants of white ethnicity and aged between 40 and 71 years. 13 of the 16 interviewees were either active or moderately active according to the GP Physical Activity Questionnaire (GPPAQ).

**Results:**

Interviewees discussed a range of reasons for non-participation. These included beliefs surrounding the personal relevance of the trial based on preconceptions of intervention content. Many interviewees considered themselves either sufficiently active or too functionally limited to increase activity levels further, so rendering participation pointless in their view. Other identified barriers included a lack of free time, for trial participation and for increasing physical activity, and dissatisfaction with appointment scheduling systems in place at GP surgeries. Interviewees questioned the appropriateness of primary care as a context for delivering interventions to promote physical activity. In general, interviewees were positively disposed towards the idea of trial participation, especially if personal benefits are made salient, but suggested that interventions could be delivered in a different setting such as the internet.

**Conclusions:**

To increase participation in physical activity promotion trials conducted in primary care, the content of invitation materials and procedures for contacting potential participants require reconsideration. Specific recommendations include streamlining intervention materials and enhancing their relevance to the health concerns of invitees.

**Trial registration number:**

ISRCTN72691150; Pre-results.

**Strengths and limitations of this study**

- Research into reasons for non-participation in primary care-based physical activity trials is lacking. This study recruited a difficult-to-engage group in qualitative telephone interviews, allowing for in-depth exploration of influences on their decisions not to take part.
- Our interviews revealed a number of novel themes, including interviewees’ beliefs that primary care may not be the most favoured setting for delivery of preventative health advice given commonly held beliefs that services are already overstretched and treatment for existing health conditions should be prioritised.
- Our extremely low interview uptake rate suggests that a wider range of themes may emerge from interviews with more diverse sample groups, although we remain unsure how best to increase recruitment of these groups.
- We may have replicated the sample non-representativeness that we wished to explore in our interviews by employing the same postal recruitment strategy used for the Very Brief Interventions trial. Using alternative means of recruitment may increase interview participation and broaden the type of interviewees recruited.
- We acknowledge that we were unable to recruit any Caucasian or unemployed non-participants, suggesting that themes specific to these groups are unlikely to be touched on in our interviews.

## Introduction

Poor representativeness of research study samples is a well-evidenced problem that can limit our ability to generalise research findings to the wider populations from which study samples are initially derived.^1^ Where randomised controlled trials (RCTs) are conducted to assess the efficacy of public health interventions, generally considered the ‘gold standard’ in study design, strong internal validity (correct and unbiased assessment of the association between an intervention and an effect) may be ensured at the expense of maximising external validity (the ability to generalise findings to other populations).^2^ ^3^

Trials of interventions to promote physical activity are a case in point, with participation shown to vary according to an individual's sociodemographic characteristics, such as their gender, ethnicity, education levels, living circumstances and income status.^4^ ^5^ This scenario is a cause for concern for those keen to ensure that public health interventions do not inadvertently exacerbate existing inequities in health (defined as inequalities in health deemed unfair or stemming from social injustice),^6^ given that a number of these characteristics are also markers for social disadvantage. Poorer health behaviours, including physical inactivity, are understood to cluster in disadvantaged groups, and it may contribute to the less favourable health outcomes that these groups more often experience.^7^

As a result, we have now reached a situation in which our best available evidence on how to promote physical activity across a population is derived from research conducted in samples of the least deprived, healthiest or most active individuals in that population (ie, healthy volunteer bias).^4^ ^5^ ^8^ One implication of this may be that interventions proven efficacious in research trials subsequently demonstrate lower effectiveness when rolled out to more heterogeneous populations, potentially performing the worst in groups already at higher risk of ill health due to social disadvantage, thus exacerbating existing differentials in health.^3^

To overcome this problem, actions are required to improve study sample representativeness. To achieve this goal, we first require a better understanding of the reasons why individuals do not take part in these trials. If potentially modifiable barriers to participation are identified, we may then be in a position to remove them. Existing quantitative research in this area has identified a diverse range of possible influences on decisions to participate in physical activity interventions, with comprehensive overviews of this topic available in systematic reviews conducted by Foster *et al*^4^ and Pavey *et al*.^9^ The present study contributes to the existing literature exploring determinants of physical activity trial uptake using qualitative methods.^5^ ^8^ ^10^ A qualitative approach is advantageous here as it allows us to gain a deeper understanding of the motives underlying non-participation.

The present study differs from existing qualitative investigations on this topic as it recruited and explored the views of a group of physical activity trial non-participants directly. While a number of published studies have examined influences on participation using qualitative methods either in trial participants themselves or with target groups residing in communities of interest,^11–13^ we were able to identify just one that interviewed non-participants to a physical activity trial specifically.^14^ That study focused on older individuals, and reported influences on participation including aspects of trial design and delivery, interviewees’ current health and activity practices and practical factors such as available free time. Beyond considering the characteristic of age, this work did not directly discuss the potential impact of other biases in uptake on intervention equity.

The present study aimed to interview a sample comprising a wider age range, and follows on from a prior quantitative analysis comparing the sociodemographic characteristics of participants and non-participants to an earlier pilot physical activity trial.^15^ This analysis demonstrated greater likelihood of participation with increasing age, but also that the general practice (GP) surgery at which patients were registered played an apparently larger role in explaining decisions to participate than individual demographic characteristics. Given data collection limitations pertaining to this pilot study, we were unable to explore within this previous analysis exactly which aspect of the GP surgery influenced invitees’ the most. Qualitative interviews of non-participants to a subsequent main randomised controlled trial were thus proposed as a means to follow-up this issue and explore possible influences in greater depth.

The aims of the present study were to identify reasons that non-participants to a primary care-based physical activity trial give for not taking part, to determine whether these reasons relate to aspects of the research trial procedure or to the intervention content and to understand more about if or how they contribute to poor study sample representativeness. Finally, we wished to derive directly from non-participants a list of recommendations for enhancing uptake into future trials in this area.

## Method

### Setting

We interviewed a sample of GP registered patients who were invited, but declined to participate in the ‘Very Brief Interventions’ (VBI) RCT. Recruitment into this trial is currently underway and is being conducted alongside recruitment into England's National Health Service (NHS) ‘Health Check’ programme, predominantly using postal invitations from October 2014 to December 2015. Inclusion criteria for the trial were identical to those of the Health Check (box 1).

Box 1 **The Very Brief Interventions (VBI) trial and National Health Service (NHS) Health Check**

The VBI trial is a primary care-based randomised controlled trial examining the effectiveness and cost-effectiveness of a very brief pedometer-based intervention (ie, delivered in ∼5 min) to promote physical activity.

The VBI trial was delivered within the context of the NHS Health Check programme. The Health Check screening initiative invites patients in the age range of 40–74 years without a prior history of vascular disease (type 2 diabetes, heart disease, kidney disease or peripheral vascular disease) to attend a screening appointment at their general practice surgery. During the screening appointment, patients undergo cholesterol and blood pressure tests, assessment of their body mass index and risk of type 2 diabetes. Patients are subsequently offered medical intervention or lifestyle advice, delivered by a health professional, as required based on their test results.

### Participants and procedures

Non-participants eligible for interview were identified from GP surgery records as patients who had been sent a VBI trial invitation and two subsequent reminder invitations, but who did not book a Health Check and enrol into the VBI trial within 4 weeks of the last reminder mail-out. Unless they had actively opted out of receiving further contact from the study team, participants were posted another invitation to complete a research interview via telephone.

Invitees who were interested to participate contacted the study team directly via prepaid return postcard, supplying their contact details and availability, and were telephoned by the primary researcher (SA) to schedule an interview slot. Where individuals were not contactable by telephone after four attempts, a maximum of two emails or letters were then sent. We aimed to recruit a sufficient number of non-participants to reach saturation of emerging themes. Cognisant of the likely difficulties involved in engaging this group, we opted for convenience sampling of willing individuals. A £20 reimbursement was offered to participants for their time and in hope of encouraging uptake of interviews based on existing evidence that cash incentives may enhance participation of hard-to-reach groups.^1^

Between April and August 2015, a total of 955 invitations were sent to trial non-participants registered at five GP surgeries in the East of England, UK. Details of participating GP surgeries are provided in table 1. Invitations stated that the primary purpose of the interviews was to explore reasons for non-participation in a research study that aimed to promote physical activity. We have no record of how many sent invitations actually reached potential recipients as initial mail-outs were managed entirely by GP surgery staff, with no study team involvement permitted during initial contact stages. No further record of the numbers of individuals responding to initial and reminder mail-outs was kept.

**Table 1:** Details of collaborating GP surgeries

GP surgery	Registered persons	Location*	Practice index of multiple deprivation, score and decile†	Practice % black or ethnic minority patients	Number of interviewees recruited
Practice 1	7432	Urban city and town	22.0 (5th decile)	3.5	2
Practice 2	9727	Urban city and town	22.3 (5th decile)	3.1	3
Practice 3	9297	Urban city and town	20.1 (6th decile)	10.7	1
Practice 4	9775	Urban city and town	14.3 (8th decile)	17.2	8
Practice 5	9208	Rural town and fringe	8.1 (10th decile)	5.1	2

*Based on GP LSOA classification.

†Data (2015) derived from Public Health England General Practice Profiles.^16^ Decile of deprivation, UK ranking (2015). 1=least deprived, 10=most deprived.

GP, general practice; LSOA, lower super output area.

Twenty-five non-participants contacted the study team expressing an interest in being interviewed. This led to 16 completed interviews. On average, participants required two contact attempts to schedule an interview, with a minimum of one telephone call and a maximum of four calls plus two email follow-ups. Reasons for non-completion of interviews in the nine initially interested parties included inaccurate contact details (N=4) or an inability to reach the individual via phone, email or post following the maximum number of contact attempts (N=5). Prior to the interviews, each interviewee returned a signed consent form to the study team via post.

Interviews were conducted by the primary researcher (SA), a female PhD student in behavioural science who had previously undertaken qualitative methods training. Interviews lasted between 20 min and 1 hour, with an average duration of ∼30 min. Verbal consent for the audio recording of interviews was obtained and recorded at the start of each interview. Notes of any relevant observations were taken during the interview.

### Materials

An interview topic guide (box 2) was developed to explore reasons for trial non-participation, more general views on health, participation in health interventions via primary care and current physical activity practices. Questions were phrased to elicit open-ended answers from interviewees, with additional probes designed to encourage elaboration on responses. The topic guide was pilot tested for coherence and relevance among the study team prior to interviews commencing.

Box 2 **Interview topic guide**

 1. Memory of the main randomised controlled trial invitation letter and views on this letter.

 2. Primary stated reason for non-participation in the Very Brief Interventions (VBI) trial (and the National Health Service Health Check).

 3. Expectations of what trial participation would involve.

 4. Attendance at the Health Check appointment and if the interviewee considered the Health Check and VBI trial to be separate interventions.

 5. Understanding of vascular disease, risk factors and personal susceptibility.

 6. Understanding the role of physical activity in the prevention of vascular diseases.

 7. Current physical activity practices, influences on physical activity and sources of information on physical activity.

 8. Views of and main influences on current health state.

 9. Previous participation in health interventions or trials, including those that are physical activity specific.

10. Views on general practice surgery and on physical activity intervention and trial delivery in this setting.

11. Recommendations on improving invitation procedures and on the design of physical activity interventions or trials.

This topic guide was informed by results of the prior quantitative analyses conducted using data from VBI pilot trial participants.^15^ We adapted the topic guide in an iterative manner over the course of interviewing to maximise utility. Directly following the interview, interviewees filled out a demographic questionnaire and a brief self-report physical activity questionnaire (GPPAQ), also via telephone.^17^ Brief notes were made during interviews of any comments considered potentially helpful in aiding interpretation of interview transcripts (eg, further information on demographic characteristics or physical activities commonly performed).

### Analysis

Audio recordings were transcribed verbatim by an independent transcription service, and text files imported into NVivo V.9 (qualitative analysis software). We conducted descriptive thematic analysis, involving initial familiarisation with interview transcripts through repeated reading, line-by-line coding to identify emergent themes and subsequent linking of these into higher order themes. A data-driven, descriptive approach was chosen, rather than using a priori codes to interpret the data, as we intended to capture and describe the range of views expressed by interviewees rather than explore in greater depth a more limited set of preidentified themes as dictated by the existing evidence base.^18^ Our analysis intends to offer those who may be considering conducting physical activity interventions some insight into potential barriers to participation and to derive some pragmatic recommendations on how such issues may be overcome. Coding was performed independently once by the first author (SA) for all transcripts, with 25% of transcripts independently coded by the second author (KLM). The final set of themes was decided on and clarified through discussion between these two authors.

## Results

Table 2 provides demographic details of the interview sample. All interviewees were of white ethnicity, aged between 40 and 71 years and were either in employment or retired. We interviewed more female than male non-participants, and the sample was, on average, more educated and earning higher salaries than the UK average. The majority of interviewees were classified as either active or moderately active according to the GPPAQ. Our interview sample was more similar to prior VBI pilot trial participants than to non-participants in terms of ethnicity and gender composition, but was of similar mean age to VBI pilot trial non-participants. As the main VBI RCT is currently underway, no summary of the demographic characteristics of participants and non-participants is, as yet, available. These comparisons suggest that our interview sample may under-represent Caucasian, male and inactive non-participants. We therefore acknowledge that we are unlikely to have reached saturation of themes based on the limited number of interviews conducted.

**Table 2:** Interview sample characteristics

	Interview sample	Health Check and VBI pilot trial non-participants*	Health Check and VBI pilot trial participants*
Demographic characteristics			
Ethnicity			
White	16 (100%)	585 (69.3%)	163 (84.0%)
Gender			
Female	10 (63%)	417 (49.4%)	114 (58.8%)
Male	6 (37%)	427 (50.6%)	80 (41.2%)
Age (mean years, SD)	52.2 (10.0)	52.0 (9.0)	54.4 (9.3)
Employment		No information available	
Paid work	11 (69%)		
Homemaker/parent	0 (0%)		
Unemployed	0 (0%)		
Retired	4 (25%)		
Student	1 (6%)		
Manual	5 (45%)		
Non-manual	6 (55%)		
Education			
None	1 (6%)		
GCSE or similar	6 (38%)		
AS/A level or similar	1 (6%)		
Degree similar	8 (50%)		
Other	0		
Household income			
<£18 000	3 (19%)		
£18 000–£30 999	5 (31%)		
£31 000–£51 999	5 (31%)		
£52 000–£100 000	2 (13%)		
>£100 000	1 (6%)		
Marital status			
Single	3 (19%)		
Married	7 (44%)		
Separated/divorced	3 (19%)		
Widowed	0 (0%)		
Cohabiting	3 (19%)		
Physical activity index (GPPAQ)			
Active	10 (63%)		
Moderately active	3 (19%)		
Moderately inactive	1 (6%)		
Inactive	2 (13%)		

Values are given as N (%).

*Data derived from a quantitative analysis of VBI trial recruitment.^15^

AS/A, Advanced Subsidiary level/A level; GCSE, General Certificate of Secondary Education; GPPAQ, General Practice Physical Activity Questionnaire; VBI, Very Brief Interventions.

Across the 16 interviews, we identified six overarching influences on decisions not to participate. These are a misunderstanding of the invitation, lack of perceived relevance, time constraints, GP surgery-related barriers, views on research trial participation and recommendations for increasing uptake.

### Understanding of the invitation process

Our initial questions probed views on the invitation process (the original invitation letter is available in online supplementary file 1 and the participant invitation sheet in online supplementary file 2). The majority of interviewees only vaguely recollected receiving letters and did not appear to pay attention to their content. A very small number of interviewees appeared to recognise that the VBI trial and NHS Health Check were two distinct components, with the possibility to attend the Health Check without trial participation. The remainder of interviewees expressed some uncertainty as to what exactly they were being asked to participate in (box 3).

10.1136/bmjopen-2016-011577.supp1 Supplementary data

10.1136/bmjopen-2016-011577.supp2 Supplementary data

Box 3 **Misunderstanding of the invitation**

“I did read through them all, but I didn’t seem to digest it”. (P12, men, 44 years, active)

“Just a health check. I don't really know what it would have involved”. (P14, women, 41 years, active)

“I can't actually remember it, to be honest, but I probably didn't think about it that much”. (P1, women, 70 years, active)

### Perceived lack of personal relevance of the trial

On receipt of the invitation letter, interviewees appeared to form expectations of what the intervention would involve and, based on these, decided whether they were appropriate ‘candidates’ for participation (box 4). A number of interviewees expressed a belief that they were not the type of person that we, as researchers, were seeking, largely because they either self-identified as already sufficiently active or felt that the information they would receive would be of no use to them. Results from the GPPAQ questionnaire generally corroborated interviewees' self-identified ‘active’ status (ie, 12 of 13 active or moderately active interviewees correctly labelled themselves as so; box 2). Most interviewees were aware of the existence of physical activity guidelines for their age group, although few were confident in their knowledge of what these entailed or how increasing activity might benefit health.

Box 4 **Non-candidacy based on current activity or weight status**

“I already do run about four times a week and cycle to and from work…and do Pilates, so I probably wouldn't increase it. I felt like probably I wasn't the ideal candidate”. (P15, women, 43 years, active)

“Not being disrespectful…I know what I should do for myself”. (P5, women, 42 years, moderately inactive)

“Maybe like 20 minutes five times a week or something. I can't remember, maybe half an hour”. (P14, women, 41 years, active)

“I would link together obesity and lack of physical exercise, and like I say, luckily I'm quite a tall slim bloke, you know, it's never been a concern for me”. (P11, men, 57 years, active)

“I’d like to think I could maintain the level of health I’ve got and my functionalities. I’d like to remain an active person”. (P2, men, 45 years, active)

When considering participation, interviewees tended to take into account their current activity levels, as well as looked to their body as a signifier of candidacy. Nearly all interviewees commented unprompted on their weight or shape during the course of the interview, citing this as a key influence on their activity habits. Where losing weight was not given as a prominent motive for increasing physical activity, interviewees mentioned the importance of activity for retaining physical functionality or for slowing down the physical degeneration that they anticipated would accompany the ageing process.

For a smaller number of interviewees, decisions on perceived relevance were based on opposite reasoning; namely, that an existing health condition or physical disability, whether temporary or permanent, rendered them unable to increase activity levels (box 5). In spite of this, interviewees were generally positively disposed towards the idea of participating in the trial, but highlighted that intervention content would need to be sensitively tailored to the physical limitations that they faced.

Box 5 **Non-candidacy based on a health condition or disability**

“I think it's a good idea but I don't know if it would be a good idea for me. I mean, I've got arthritis”. (P4, women, 53 years, inactive)

“If I could do more exercise, it would be great, but exercises that we could manage, I could physically manage, so I could manage the pain with it”. (P3, men, 48 years, moderately active)

“I'd been down the health centre a lot that month and I just thought oh no, I can't do that again. I don't want to go down there again”. (P6, women, 62 years, active)

“Having had so much time off already, so yes that was another issue really”. (P6, women, 62 years, active)

“Because I've got so many medical problems, I didn't really want to find out if I've got anything else wrong with me”. (P4, women, 53 years, inactive)

“With all the checks I had I would suppose that I'm quite healthy”. (P11, men, 57 years, active)

For interviewees with a pre-existing health condition necessitating considerable contact with the health service, the idea of attending primary care for further testing was a deterrent. Some interviewees wished to avoid the hassle of organising another appointment or finding additional time out to attend, while others expressed anxiety about the potential for new tests to reveal yet more health problems. A few interviewees who had recently undergone extensive testing for a pre-existing but unrelated health condition also believed that they had received all necessary tests to confirm that their current health was good, and so did not consider themselves at risk of vascular disease or in need of further examination.

### Time constraints

Time constraints were frequently cited as barriers to participation, generally construed as resulting from external forces beyond individual control, and were often offered as justifications for non-participation at the start of interviews prior to even being questioned on this matter (box 6). Work and/or family commitments were most commonly expressed as reasons for limited time, in terms of an inability to participate and more generally precluding increasing physical activity levels.

Box 6 **Time constraints and prioritisation of prevention**

“In all fairness there was no decision not to attend, I wanted to do it…and then things get put to one side…I was busy with work”. (P12, men, 44 years, active)

“I work nine to five in an office and then come back home and I've got two children and my husband…. So then by the time I finish with them it will be like half past eight or eight o'clock and then I feel too tired to…”. (P5, women, 42 years, moderately inactive)

“I'll be honest with you, at the time it wasn't actually on my list of priorities”. (P2, men, 45 years, active)

“You just think, well, actually if I was overweight and I'm not exercising now and eating the wrong foods then obviously…so for me I felt that, yeah, it would just be a little bit patronising”. (P8, women, 40 years, inactive)

When further probed to elaborate on daily routines, a number of interviewees acknowledged that low prioritisation of preventative health interventions may be less of an influence on non-participation than time constraints. Where current health or health behaviours were considered good, the lack of any pressing need meant that many interviewees did not necessarily see value in allotting time to receiving or acting on advice to increase activity levels.

### GP surgery-related barriers

A number of interviewees expressed surprise at the idea of their GP surgery providing advice on physical activity. Interviewees tended to view their surgery as a place to go only when already sick. Concerns about taking up limited staff time to discuss health prevention were expressed, with this seen as a less legitimate use of resources than attending primary care for treatment of an existing condition (box 7). For some interviewees, the provision of advice on physical activity was something that would only be welcomed if relevant to an existing health condition. Some interviewees were keen to avoid being dictated to or patronised by health professionals giving information that interviewees were already familiar with.

Box 7 **General practice (GP) surgery-related barriers to participation**

“So, you know, unless I've got any particular issues then I don't usually see the GP at all”. (P7, men, 58 years, moderately active)

“The fact is that to me a doctor is somebody you go and see when you’re sick, and you're not feeling well. If you’re generally feeling well you don’t always consider going and taking up their time to find out if there is any way you can improve your health. It just doesn’t sit right, you know…”. (P2, men, 45 years, active)

“I'd be quite happy to receive any advice, you know, if I went to the doctor with a problem and it was attributed to lack of, you know, physical exercise”. (P11, men, 57 years, active)

“You think, oh gosh, they're telling me how to live my life sort of thing. I think sometimes it can feel a bit dictatorial when somebody's telling you things that you haven't gone to seek out”. (P16, women, 71 years, moderately active)

“I find it very frustrating as a busy, full-time person. I cannot make an appointment to see my GP other than on the very day”. (P15, women, 43 years, active)

“But just no one would pick the phone up at the surgery for me to actually book on”. (P3, men, 48 years, moderately active)

A common theme across interviews was an expression of frustration with accessing GP surgeries more generally, with many interviewees dissatisfied with appointment scheduling systems and reception staff. Notably, four of the interviewees did in fact attempt to take part in the trial, but were prevented from doing so as they could not reach their GP surgery by telephone or book a Health Check appointment during the recruitment period.

### Views on research trial participation

A number of interviewees appeared to consider themselves ‘participants’ given their involvement in the interviews, with few clearly distinguishing between the differing purposes of the original VBI RCT (testing the effectiveness of a brief physical activity intervention), the Health Check (a preventative screening initiative incorporating activity advice) and the interviews (determining reasons for non-participation).

When questioned about views on participating in physical activity research more generally, interviewees discussed weighing up potential personal and/or wider social benefits of taking part (box 8). Personal benefits included the expectation that the trial would involve some form of check on current health status or receiving novel advice on physical activity. Social benefits included a desire to give back to others or seeking recognition or praise for participation. This may be recognition either from future beneficiaries of research findings or from interviewees’ significant others who may have encouraged participation in the first place. When discussing these benefits, it appeared that personal gains were more relevant motivators for participation in preventative interventions generally, whereas perceived social benefits were more salient influences on the decision to participate in a research trial specifically.

Box 8 **Perceived benefits of research participation**

“I mean it's just verification would be a good way of looking at it, that what I'm doing is good for me”. (P2, men, 45 years, active)

“Usually you get some tips from research, from doctors”. (P1, women, 40 years, active)

“If people do these trials and something good comes out of it, whether it be a little bit or a big bit, you know, every little bit helps I suppose”. (P17, men, 69 years, white)

“You'd make that person feel that you done this, it's your input that helped”. (P3, men, 48 years, moderately active)

“I didn't think about it until I spoke to the wife the other day, but she said, what you tell this lady could help somebody else in the future…but I didn't look at it like that, because sometimes you don't always get it, you look at it from your own selfish point of view”. (P3, men, 48 years, moderately active)

Measurement forming part of trial protocols was generally seen as useful by interviewees who considered themselves in good health, and so expected positive feedback. However, a number of interviewees did express concern that measurement, and participation more generally, may be daunting and potentially embarrassing. Three interviewees were prior participants to another large-scale research study conducted from the University of Cambridge (the Fenland Study),^19^ due to a number of the GP surgeries from which we recruited having previously been involved in this work. Interviewees who had taken part in this past study generally reported positive experiences of the research process (box 9). Given the fact that these interviewees were prior physical activity study participants, we acknowledge that the views they express are unlikely to closely reflect those of individuals who choose to never participate in health research, thus limiting the scope of this analysis (a problem inherent in all studies of non-participant groups).

Box 9 **Research process involving measurement and feedback**

“I think if I was sure that it wasn't going to make me feel that I was inadequate in some way.” (P16, women, 71 years, moderately active)

“It was really interesting to do and, as I say, they gave you a full pack at the end of it of your measurements and details and your activities and stuff”. (P13, women, 53 years, active)

### Recommendations for increasing uptake in future

When asked for their recommendations on how invitation letters may be adapted to encourage participation, interviewees proposed that these be shortened and made more concise, with potential benefits of participation clearly highlighted. A number of interviewees also mentioned that alternative means of initial contact would be preferable, for example, email or text messaging (box 10).

Box 10 **Recommendations for increasing uptake**

“I think you need to get your message much more quickly out at the top and be much more concise quite simply”. (P10, women, 60 years, active)

“I think these days it's a bit easier if people email me to be honest with you”. (P2, men, 45 years, active)

“I do remember not being quite clear of how much time was actually involved”. (P6, women, 62 years, active)

“Internet is probably your best bet, yeah, or is what I use the most, because it's handy, because I haven't got to go out to, you know, access it”. (P3, men, 48 years, moderately active)

“I would just go and speak to people or, I mean I know some people who go to the gym three of four times a week, you know…”. (P17, men, 69 years, active)

“I get that information, oh all over the place, the Guardian newspaper, I guess, magazines, online”. (P10, women, 60 years, active)

Outlining the exact time requirements of participation was also suggested as a useful addition to invitation materials. When asked to offer further recommendations on how we may overcome time constraints, interviewees suggested extending recruitment deadlines or offering more flexible appointment times or settings. More generally, non-participants tended to express a view that primary care is not necessarily the most appropriate setting for physical activity promotion. Interviewees reported using the internet, television, print media or more physically active friends as their most common and favoured sources of information and norms regarding physical activity.

## Discussion

Through qualitative interviews with physical activity intervention non-participants, we found that decisions to not take part were influenced by interviewees’ beliefs surrounding the personal relevance of the intervention, which appeared to be based on often inaccurate views of what they would be required to do. Other identified barriers included perceived lack of free time, for attending a Health Check appointment and for increasing physical activity, dissatisfaction with appointment scheduling systems in place at GP surgeries and a view that primary care may not be the most appropriate setting for the delivery of health promotion interventions.

Certain of these themes have conflicting implications for the issue of study sample representativeness and its impact on physical activity intervention equity. Our interviews suggest that the most active and the most physically limited or unwell are opting out. Non-participation in the latter group may mean that conclusions drawn from research trials in this area do not necessarily generalise to patients suffering from function-limiting health conditions. In some cases, self-exclusions may be warranted if an existing health condition contraindicates increasing activity, but in many other cases, these patients may in fact benefit from becoming more active. Clearly communicating the potential risks and benefits of trial participation for people who have a pre-existing health condition or physical limitation during the invitation process may help to promote uptake in those currently unsure of their candidacy.

The opposite situation identified through our interviews, of apparently active individuals also not participating, may be less of a concern with regard to sample non-representativeness leading to the development of interventions with the potential to increase inequities in physical activity levels. This does, however, depend on the extent to which individuals can accurately identify whether or not they meet physical activity guidelines. Although the majority of interviewees who considered themselves active were confirmed as such using the GPPAQ, our lengthy discussions of activity during the interview are likely to have primed responses to this questionnaire, potentially inflating activity reports.^20^ When probed, we found that many interviewees were unclear as to how much activity should be performed in order to benefit health, a finding supported by existing quantitative research showing that individuals may be relatively inaccurate judges of their own activity levels^21^ ^22^ and that confusion as to what constitutes ‘physical activity’ is commonplace.^14^ ^23^ Nearly all interviewees were unable to explain how physical activity can reduce vascular disease risk, aside from its impact on body weight. This suggests that including information on the proposed mechanisms that underlie physical activity's preventative effects for a specific condition of interest, already a recommended behaviour change technique for promoting activity in the context of primary care,^24^ may also prove a useful addition to invitation letters.^25^

Communicating this information may also help to overcome one further stated barrier to participation, namely the low priority assigned to preventative health interventions. That this theme emerged from our interviews suggests that potential benefits of health promotion are still not communicated effectively enough despite the long history of delivering such interventions within primary care. This point is further supported by comments from a number of interviewees indicating that preventative interventions are in some cases seen as detracting GPs away from time better spent treating pre-existing health conditions in what is perceived as an already overburdened health system. It would appear that more careful consideration now needs to be given to how best to highlight the potential direct health-related and service-related benefits that may be derived from widespread participation in programmes designed to maintain and improve current health states, for example, including advertising or posters highlighting the benefits of prevention in GP waiting rooms.

Remaining themes identified through our interviews corroborate those identified in existing qualitative research exploring determinants of uptake of physical activity trials and preventative health interventions more generally (including to the NHS Health Check specifically). These include perceptions of the inappropriateness of the intervention to current health status,^26^ negative prior experiences in primary care,^12^ ^27^ time constraints,^13^ beliefs surrounding the anticipated benefits of taking part^28^ and the aforementioned low prioritisation of health prevention.^11^

The extent to which such barriers may lead to recruitment of non-representative research samples is likely to be contingent on the degree to which their expression is sociodemographically patterned. Existing evidence indicates that groups at greatest risk of social disadvantage, defined according to a number of indicators (eg, lower socioeconomic status, older age, female gender, those experiencing physical or mental disabilities, among other groups), are more likely to be in poorer health^29^ and to receive less satisfactory healthcare,^30^ meaning that they may be more likely to vocalise themes relating to these experiences. This might also be true of the theme of time constraints as a barrier to participation and to increasing physical activity, given research demonstrating that certain disadvantaged populations more commonly report inflexibility in working schedules,^12^ greater childcare demands^31^ or transport limitations that increase the inconvenience of accessing activity facilities.^32^ Future research exploring barriers to participation that specifically compare the experiences of individuals facing differing levels of disadvantage in order to more clearly map barriers that may be considered generic versus those that are population subgroup specific and so require targeted efforts to surmount would prove useful to explore this issue further. We acknowledge that the views expressed by our sample, comprising non-participants willing to complete interviews (2.7% of all individuals asked to participate), are unlikely represent opinions held by all non-participant groups. This fact is additionally supported by considering the sociodemographic profiles of interviewees, shown in table 2, which suggest that they may be more similar to prior VBI pilot trial participants than non-participants, with a clear lack of racial heterogeneity. We welcome further work exploring determinants of non-participation conducted in more diverse interview samples (see recommendations below on how to engage these groups).

### Recommendations for increasing participation rates

The majority of interviewees considered our original invitation letter and participant information sheet to be too lengthy and containing the wrong type of information to allow for accurate understanding of what participation exactly entailed. Moreover, the fact that original invitation letters to the NHS Health Check and to the VBI trial were sent together in a single mail-out may have added complexity to the recruitment process, leading recipients to conflate the specific aims of each component. Their recommendations to shorten invitation materials has obvious implications for the ethics approval process, with ethics committees generally requiring specific details to be incorporated into these communications, thus contributing to their length.^33^ Comments from interviewees suggest that a review of how this information can be better presented is now timely, as is the conduct of further embedded trials of optimised recruitment materials (eg, refer Man *et al*).^34^

Interviewees’ critiques of primary care as a context for physical activity promotion were clear and suggest that there is much room for improvement in the design and delivery of trials in this setting. Given that a number of interviewees suggested that advice to increase activity may only be welcomed if relevant to an existing health condition, opportunistic physical activity promotion during routine primary care consultations may represent a plausible alternative approach. For this to succeed, buy-in from primary care practitioners is obviously required, potentially facilitated by the provision of relevant training materials that highlight the value of activity for disease prevention, in addition to offering practitioners a range of practical techniques on how to deliver brief but effective physical activity interventions to patients they consider eligible beneficiaries.^10^ ^35^ Future work may also benefit from exploring possible differences in the acceptability and effectiveness of preventative health interventions delivered by members of practice staff other than general practitioners, or by external staff (eg, health promotion specialists), thereby potentially circumventing the stated barrier of not wanting to absorb general practitioner time in discussing long-term prevention.

Considering alternative contexts for intervention was another recommendation from interviewees, many of whom favoured the internet as a source of information on health behaviours and as a favoured mode of invitation via email. Given the potential of internet-based interventions to facilitate rapid tailoring of content to specific user groups, this technology is likely to prove useful as a means to enhance the relevance of a physical activity intervention to potential participants. This relevance, if clearly communicated in invitation materials, may go some way towards promoting uptake in those who might otherwise consider themselves inappropriate candidates for more generic interventions in which personal benefit remains unspecified. Interventionists aiming to invite participants or to deliver health prevention programmes via the internet do however need to remain mindful of social patterning in the access to and preferences for using this technology,^36^ and ensure that sole reliance on online resources does not lead to systematic exclusion of certain population subgroups who may have less opportunity to access or experience in using the internet. One further consideration, given the rapid proliferation of online and mobile health interventions, pertains to the relative effectiveness of internet-based versus face-to-face delivery of physical activity interventions. We were able to locate just one review available comparing these interventions settings, itself comprising a single trial that identified no significant differences in effect.^37^ More work is now needed to determine whether delivering physical activity interventions via these newer platforms can elicit changes in behaviour of a similar magnitude to that obtained in more traditional settings, thereby ensuring that any potential improvements in uptake are not offset by reductions in intervention effectiveness.

## Conclusions

Perceptions of the relevance of a primary care-based physical activity trial to invitees’ current health status or level of physical functioning influenced decisions to participate, and may lead to recruitment of study samples that are not necessarily representative of wider primary care patient populations. To ensure that physical activity promotion interventions conducted in this setting produce equitable increases in activity, we need to improve the generalisability of trials informing their content. Addressing non-participants’ criticisms of invitation procedures and finding innovative solutions to overcome stated barriers to participation may be the first step towards achieving this goal, with the tailoring of invitation materials delivered via the internet a potential avenue for further exploration.
