# Supplementary material for: Reasons for non-participation in a primary care-based physical activity trial: a qualitative study
Source: BMJ Open. 2016 May 23;6(5):e011577. doi: 10.1136/bmjopen-2016-011577 (PMC4885436; doi:10.1136/bmjopen-2016-011577)
Supplement: Supplementary data [file bmjopen-2016-011577supp1.pdf]

PRACTICE HEADED PAPER

PRACTICE ADDRESS
PRACTICE PARTNERS

[Date]

<insert name of participant>
<insert address line 1>
<insert address line 2>
<insert address line 3>
<insert postcode>

Dear *<insert name of participant>*

The VBI Trial: A Randomised Controlled Trial of the efficacy and cost effectiveness of a Very Brief Intervention to increase physical activity among adults attending NHS Health Checks

We are currently working with The Primary Care Unit at the University of Cambridge on a research project entitled the ‘Very Brief Interventions Study’. This research aims to develop new ways of increasing physical activity in the population by delivering very brief physical activity advice as part of the NHS Health Check.

We are writing to you because you are eligible for an NHS Health Check and we would like to invite you to take part in the trial. You will find enclosed a participant information leaflet which provides you with all the details of the study. Please read this document carefully before deciding whether you wish to take part.

You are under no obligation to participate in this trial. Should you choose not to take part, **you are still eligible to attend the NHS Health Check**.

If you would like additional information or wish to discuss the trial further, please feel free to contact the Trial Coordinator, Ms Jo Mitchell, on tel: 01223 761760 or email: VBI_Study@medschl.cam.ac.uk.

Thank you for considering our invitation to participate in this research.

Yours sincerely

<Practice Manager’s name here>

<Signature here>
