# Supplementary material for: Reasons for non-participation in a primary care-based physical activity trial: a qualitative study
Source: BMJ Open. 2016 May 23;6(5):e011577. doi: 10.1136/bmjopen-2016-011577 (PMC4885436; doi:10.1136/bmjopen-2016-011577)
Supplement: Supplementary data [file bmjopen-2016-011577supp2.pdf]

VBI VERY BRIEF INTERVENTIONS

Very brief interventions to promote physical activity in primary care

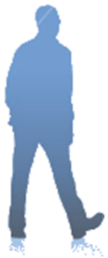

PARTICIPANT INFORMATION SHEET A

The VBI Trial

You are being invited to take part in a research trial which is part of a large programme of research. In this research programme we are looking at ways to help people become more active by providing very brief physical activity advice. This advice will last no more than 5 minutes and will be given during a routine NHS Health Check.

Please take the time to read the following information. This will help you decide whether or not you would like to take part. It is important that you understand why this trial is being done and what it will involve. Part 1 tells you the purpose of the trial and what will happen to you if you take part. Part 2 gives you more detailed information. If you have any queries or would like any advice about taking part in research please contact the trial coordinator (contact details are on the back page) who will answer your questions or put you in contact with the right person to help.

Part 1:

What is the purpose of the research?

It is often in the news that we should increase the amount of physical activity we do. Small lifestyle changes can improve our health, help us to lose weight and lower the risk for developing vascular diseases (e.g. heart disease, kidney disease, type 2 diabetes and stroke).

We are doing a randomised controlled trial (RCT) to find out whether people become more active after they have received some very brief advice on how to increase physical activity. This very brief advice is given at the end of a NHS Health Check. Health Checks are held at your local GP surgery.

In a randomised controlled trial we put people into groups and give each group a different treatment. “Treatment” for this trial is either the Health Check on its own or the Health Check with some extra advice designed to encourage an increase in physical activity. The results are compared to see if one is better. To try to make sure the groups are the same to start with, each patient is put into a group by chance (randomly).

Why have I been chosen?

You have been selected randomly (i.e. by chance) to take part because you are eligible for the NHS Health Check according to your General Practice records. Eligibility for the NHS Health Check is determined by age (between 40 and 74 years) and not having a diagnosis of a vascular disease.

Do I have to take part?

It is entirely up to you to decide whether or not to take part. If you decide not to take part, you are still eligible to attend the NHS Health Check.
If you would like to take part, your participation in this trial will be entirely voluntary. You are completely free to withdraw at any time you choose without giving a reason. This will not affect the standard of care given to you during the NHS Health Check or by your GP practice in general.

What will happen if I decide to take part in the trial?

At the beginning of your Health Check the practice nurse or healthcare assistant will ask you to sign a consent form and complete a short questionnaire. The questionnaire asks some questions about your background such as education, ethnic group and work. We are collecting this information so that we can build a profile of people who take part in research studies to compare with people who don't. You are free however to omit any questions you are unhappy with answering without it affecting your participation in the trial or with the healthcare you receive in general. Following this you will be put into one of two groups. To make sure both groups are equal in numbers you will be put into a group by chance (randomly). A computer will decide rather than the researcher, nurse or healthcare assistant which group you will be put into.

During the NHS Health Check the nurse or healthcare assistant will measure your height, weight and waist circumference. S/he will take your blood pressure and some blood to find out your cholesterol and glucose levels. S/he will also ask you some lifestyle questions such as smoking, alcohol and diet.

If you are allocated to the intervention group you will receive the standard NHS Health Check, complete a short questionnaire, receive some extra brief advice to become more active and be given a pedometer (a small device that counts steps) to take away and keep. This advice will be in addition to the standard NHS Health Check. If you are allocated to the control group you will receive the standard NHS Health Check and complete a short questionnaire.

Your consultation may be audio-recorded. This is done for a selection of NHS Health Checks to monitor the way in which the nurse/healthcare assistant delivers the physical activity advice. If your consultation has been chosen to be audio-taped, the practice nurse or healthcare assistant will ask for your consent to do this at the start of the consultation and your consent will be verbally recorded on the audio-recorder. You are under no obligation to have your Health Check recorded. If you would rather not have your Health Check recorded this will not affect your participation in the trial or the healthcare you receive in general
We will send you a text or give you a call, three months after the NHS Health Check, to arrange a suitable time for you to receive an accelerometer and two questionnaires. The accelerometer is a small device (about the size of a match-box) that measures body movement and is worn around the waist. We would like you to wear this for one week, during daytime only, and return it to us with the completed questionnaires in the freepost envelope provided. We do not want you to change what you do in that week but go about your daily activities as normal.

The questionnaires includes questions on the types of activities you have done and the health and other resources you may have used. The NHS use, time off work and physical activity expenditure questionnaire asks questions to help us find out whether the physical activity intervention given during the Health Check also has an effect on the use of NHS resources such as visits to the doctor's surgery and other personal expediture (e.g. joining fitness clubs etc.).

You will be entered into a prize draw when all the measurements have been completed. The draw will take place at the end of the trial (December 2015) and 20 participants will each receive a £20 voucher.

The purpose of the NHS Health Check is to calculate your risk of developing vascular diseases over the next 10 years. With your permission we would like to collect this information from your medical records. This information along with the rest of the information collected will help us understand why some people increase their physical activity more than others.

What are the possible risks of taking part?

We do not anticipate any risks associated with participation in the trial. The aim of the brief intervention is to promote physical activity such as brisk walking and cycling which carries a very low risk of injury or harm. Please remember that you are free to withdraw from the trial at any time without giving a reason.

What are the possible benefits of taking part?

Physical activity can delay or prevent the onset of vascular disease. Receiving advice on how to become more active could improve your health, for example by helping you to lose weight and lowering your blood pressure. Your participation could also help improve the quality of brief physical activity advice given in primary care

Will my taking part in the trial be kept confidential?

Yes, you will be assigned an ID number to which all your data will be linked. All information which is collected about you during the course of the research will be kept strictly confidential, and any information about you which leaves the surgery will have your name and address removed so that you cannot be recognised. Only the research team will be able to identify you, which is necessary for the purpose of contacting you to send the accelerometer and to send a newsletter at the end of the trial. With your consent, your GP practice will also be informed of your involvement in the trial so that this information can be included in your confidential medical records.

All data collected, including any audio-taped recordings and interview transcripts, will be stored securely, in accordance with the Data Protection Act and the Primary Care Unit's (PCU) policy on security. Paper copies will be stored in locked filing cabinets and electronic data will be stored on encrypted volumes on a University of Cambridge file server. The PCU is a card accessed building.

All audio recordings will be deleted from the recording device once they have been uploaded onto university computers. The anonymous recordings will be transferred by a password protected system to a transcription company (1st Class Secretarial services) to be typed out. At no point will you be identified from your recording or from the typed out version that is returned. 1st Class Secretarial Services adheres to NHS rules on confidentiality.

Only the research team (based at the Primary Care Unit and MRC Epidemiology Unit) will have access to the anonymised data, with the exception of auditors of the funder, sponsor or regulatory inspectors (Regulatory Authorities) if they decide to conduct an inspection to assess that the trial is being properly conducted.

Part 2:

Who is organising and funding the research?

This research trial is developed and conducted by the Primary Care Unit at the University of Cambridge, in collaboration with King's College London, the University of East Anglia and the Medical Research Council (MRC) Epidemiology Unit. The trial is funded by the National Institute for Health Research (NIHR).

Who has reviewed the trial?

All research in the NHS is looked at by an independent Research Ethics Committee to protect your safety, rights, wellbeing and dignity. This trial has been reviewed and given a favourable opinion by the **Cambridge East Ethics** committee (Ref: 14/EE/1004)

What will happen to the results of the trial?

You will be sent a newsletter that summarises the results of the trial. The results will be published in scientific journals and presented at conferences to other scientists, policy makers and health professionals. We will ensure that you are not personally identifiable as a participant.

What if there is a problem?

We do not anticipate there to be any major risks or problems associated with taking part in this trial. However, in the event of a claim in respect of any negligent harm caused as a result of taking part, standard NHS and University procedures for compensation are in place.

<u>Please retain this information sheet for your records.</u>

If at any time you decide to withdraw, we will keep any information we have already collected from you for our final analysis. If you object to this, please let us know and we will destroy it.

If you have a concern or wish to make a complaint about any aspect of the way you have been approached or treated during the course of this trial, please contact a member of the research team. They will do their best to answer your questions and if necessary consult the Chief Investigator, Professor Stephen Sutton. If you remain unhappy and wish to complain formally, please contact the Cambridgeshire PCT Patient Advice and Liaison Service (PALS) on FREEPHONE 0800 279 2535 or 01223 725588 or e-mail them at c-pct.pals@nhs.net. PALS are independent to the trial and will direct your concern appropriately. However, should you wish to complain about the NHS care you have received (such as the NHS Health Check itself), the normal National Health Service complaints mechanism is available to you - details can be obtained from your NHS Clinical Commissioning Groups.

What should I do next?

If you would like to take part in the trial and you have not already done so please make an appointment for your NHS Health Check with your GP surgery. Otherwise if you have made an appointment already just let the nurse or healthcare assistant know at the start of your Health Check. Please make sure you have given yourself enough time to process all the information before giving your consent to take part in the study.

<u>If you have any questions or concerns please do not hesitate to contact us.</u>

We encourage everyone to have the Health Check but we would also like as many people as possible to take part in the trial. Therefore, if you have not already made an appointment for your Health Check we will send you a reminder in about 2-3 weeks' time. If we don't hear from you after that we will assume you are not interested in taking part in the trial. In this case, your GP practice may send further letters to encourage you to make an appointment for a standard NHS Health Check.

Contact Details:

Ms Jo Mitchell (Trial Coordinator)
Phone: 01223 761760
E-mail: VBI_Study@medschl.cam.ac.uk
Address: Primary Care Unit, Institute of Public Health
Forvie Site,
University of Cambridge School of Clinical Medicine
Box 113 Cambridge Biomedical Campus
Cambridge.
CB2 0SR
Website: http://tiny.cc/VBIprog

Thank you for considering taking part in our study
